# Emulsion Cod Liver Oil

**Published:** 1873-12

**Authors:** Willard M. Rice


Emulsion of Cod Liver Oil.—*Willard M. Rice, Jr.*—After a series of experiments, at the request of, and assisted by, a medical friend, the writer of this has perfected the following formula, which he offers to his professional brethren, hoping that it may prove useful:

| | |
|---|---|
| Oleum Morrhuæ, . . . . . . | f℥viij. |
| Tragacanth, . . . . . . . . | ʒj. |
| Sacchar. Alb., . . . . . . | ʒiv. |
| Ol. Gaultheriæ, . . . . . . | gtt. lx. |
| Ol. Sassafras, . . . . . . . | gtt. l. |
| Ol. Amygd. Amar., . . . . . . | gtt. x. |
| Aquæ, . . . . . . . . . | f℥viij. |

The tragacanth and sugar are to be dissolved in the water and the mucilage strained. In this is to be incorporated first the essential oils and then the cod liver oil. This makes an elegantlooking emulsion, not too thick, containing fifty per cent. of the oil, and of a rather pleasant taste and smell.

Many manufacturers combine the lacto-phosphate of lime, etc., with the cod liver oil mixture, but as physicians often consider this decidedly objectionable in a medicine intended, as this is, in most cases, for continued use for a considerably protracted length of time, the author has been induced to omit it. It can be added, however, by a slight modification of the above formula.

